# Effects of environmental and anthropogenic landscape features on mule deer harvest in Nebraska

**DOI:** 10.7717/peerj.5510

**Published:** 2018-09-10

**Authors:** Bryan J. O’Connor, Nicolas J. Fryda, Dustin H. Ranglack

**Affiliations:** 1 Department of Biology, University of Nebraska - Kearney, Kearney, NE, United States of America; 2 Nebraska Game and Parks Commision, Kearney, NE, United States of America

**Keywords:** Mule deer, Hunting, NDVI, Roads, Nebraska, Terrain roughness, Canopy cover, Harvest, Hunter effort

## Abstract

Understanding the habitat use of wildlife species is important for effective management. Nebraska has a variety of habitat types, with the majority being covered by rangeland and cropland. These habitat types likely influence the harvest of mule deer *(*MD; *Odocoileus hemionus)* in Nebraska, but their specific effects are unknown, and moreover, harvest may also be influenced by the accessibility of deer habitats for hunters. We modeled which environmental and anthropogenic landscape features influenced harvest densities. Spatial analysis in a Geographic Information System was used to determine the mean values of environmental and anthropogenic landscape features at the county level. We then used a generalized linear model to determine which of those factors influenced MD harvest from 2014–2016. We found that NDVI amplitude, hunter effort, road density, terrain roughness, and canopy cover influence MD harvest in Nebraska. According to our model, MD harvest densities are significantly greater areas with NDVI amplitude ∼38, increasing hunter effort, road densities near 1,750 m/km^2^, increasing terrain roughness, and decreasing canopy cover. Understanding increased harvest densities of MD can be beneficial for wildlife managers, allowing for more efficient allocation of efforts and expenses by managers for population management.

## Introduction

Hunting is an important component of the North American model of wildlife management (Geist, Mahoney & Organ, 2001), and deer hunting has been a tradition in Nebraska since the mid-1900s. The first official deer hunting season was held in 1945 where 275 mule deer (MD; *Odocoileus hemionus*) bucks and 2 white-tail deer (*Odocoileus virginianus*) bucks were harvested (Nebraska Game and Parks Commission, 2016a). Along with the growing tradition of hunting, deer populations have also grown. During the 2015 hunting season 8,876 MD bucks and 28,505 white-tail bucks were harvested (Nebraska Game and Parks Commission, 2016a). The use of hunting allows for the Nebraska Game and Parks Commission (NGPC) to manage the deer population to help prevent disease, depredation issues, improve public safety, and sustain the population for future generations. Hunting also provides an economic boost for the state, contributing $562 million dollars in retail sales and supporting over 8,856 jobs (Nebraska Game and Parks Commission, 2016b). Therefore, hunting and hunter success are crucial for both Nebraska and its wildlife populations.

Hunter success, however, is complex and involves many variables. Reardon, Merrill & Taylor Jr (1978) found a direct positive correlation between the success of hunters and deer density. Additionally, habitat types are variable in quantity and quality, resulting in varying distribution and abundance of deer. Croplands do not provide the necessary amount of cover required by deer during winter, leading to increased home range size (Walter et al., 2009). However, when croplands are aggregated with forested areas or rangelands deer move less during these winter months. Close aggregation of these lands provides needed cover from forest or rangelands and nutrition from croplands (Williams, Quinn & Porter, 2012). Woolf & Roseberry (1998) demonstrated that the amount of forest cover had a positive linear relationship with deer densities, and that the limiting factors of the habitat (quality) restricted densities more than the fragmentation of habitat. Additionally, Mackie (1970) found that MD prefer slopes greater than 10%, especially during summer months. Preference for steeper slopes and rougher terrains likely mean more remote areas with lower road densities, and can cause greater difficulty for hunters in accessing these animals.

The accessibility of MD habitat for hunters is also a key factor in their ability to successfully harvest a MD. However, increasing hunters’ accessibility to deer habitat also increases road densities, which can cause negative impacts on habitat selection of deer. The occurrence of roads, however, can influence hunter success in an ambiguous way. Gratson & Whitman (2000) demonstrated higher hunter success for elk (*Cervus canadensis*) when roads were closed to vehicle access when compared to areas with open roads. However, Sawyer et al. (2007) found that elk habitat use was significantly reduced at road densities as low as 0.17 km/km^2^. Elk avoidance of roads occurs even outside of the hunting season (Ranglack et al., 2016), but successively increases during the archery and rifle hunting seasons (Ranglack et al., 2017). However, the type of habitat, forested or non-forested, that directly surrounded the roadway was important, as forested roadways allowed for higher road densities before the elk habitat use was affected. MD also show significant change in habitat selection with development, preferring habitats that are farther from roads (Sawyer et al., 2006).

During our research we investigated the leading factors that influence the number of MD harvested in Nebraska. The factors that we included were habitat type, hunter effort, road density, Normalized Difference Vegetation Index (NDVI), terrain roughness, urbanization, and canopy cover. While each habitat type can be beneficial in supporting MD survival needs (food, water, and shelter), the benefits provided may change depending on the season. For example, the nutritional benefits provided by croplands are year-round, but the cover provided by croplands is seasonal and unable to sustain yearly populations. Increased human activities in agricultural areas is also likely to discourage MD use of those areas. Therefore, it is likely that the more rugged and remote rangelands of the state will hold greater populations of MD. Consequently, higher populations of MD will likely produce higher harvest densities. However, the hunting access in these areas is more difficult, potentially reducing harvest densities. Therefore, we expect to see greater harvest densities in moderately developed rangelands, striking a balance between the MD habitat needs and hunter access.

## Materials and Methods

### Study site

Nebraska is divided into 93 counties, which we used as our sampling unit for examining MD harvest across the state. In the northwestern portion of the state, the land use is mainly rangeland and the southeastern portion is cropland, with a transition zone between the two. Moving from the northwest to the southeast portion of the state, the land-use slowly transitions towards croplands as the soil type transitions to silt (Nebraska Department of Economic Development, 2016). The main transition point of the state runs diagonal from the southwest corner to the northeast corner along the edge of the Sandhills and the loess mixed grass prairie. The rivers are also segregated on this diagonal plane, with the majority of the rivers being located in the southeast half of the state (University of Nebraska State Museum, 2016, Fig. 1). MD distribution in Nebraska follows a similar pattern. The majority of MD within the state are found in the northwest. However, they can be found throughout the western two-thirds of the state. Urbanization somewhat follows the same diagonal pattern as seen with the other variables, however, the eastern third of the state is far more populated than any other portion, especially around the Lincoln and Omaha areas (Nebraska Department of Economic Development, 2015).

**Figure 1:**
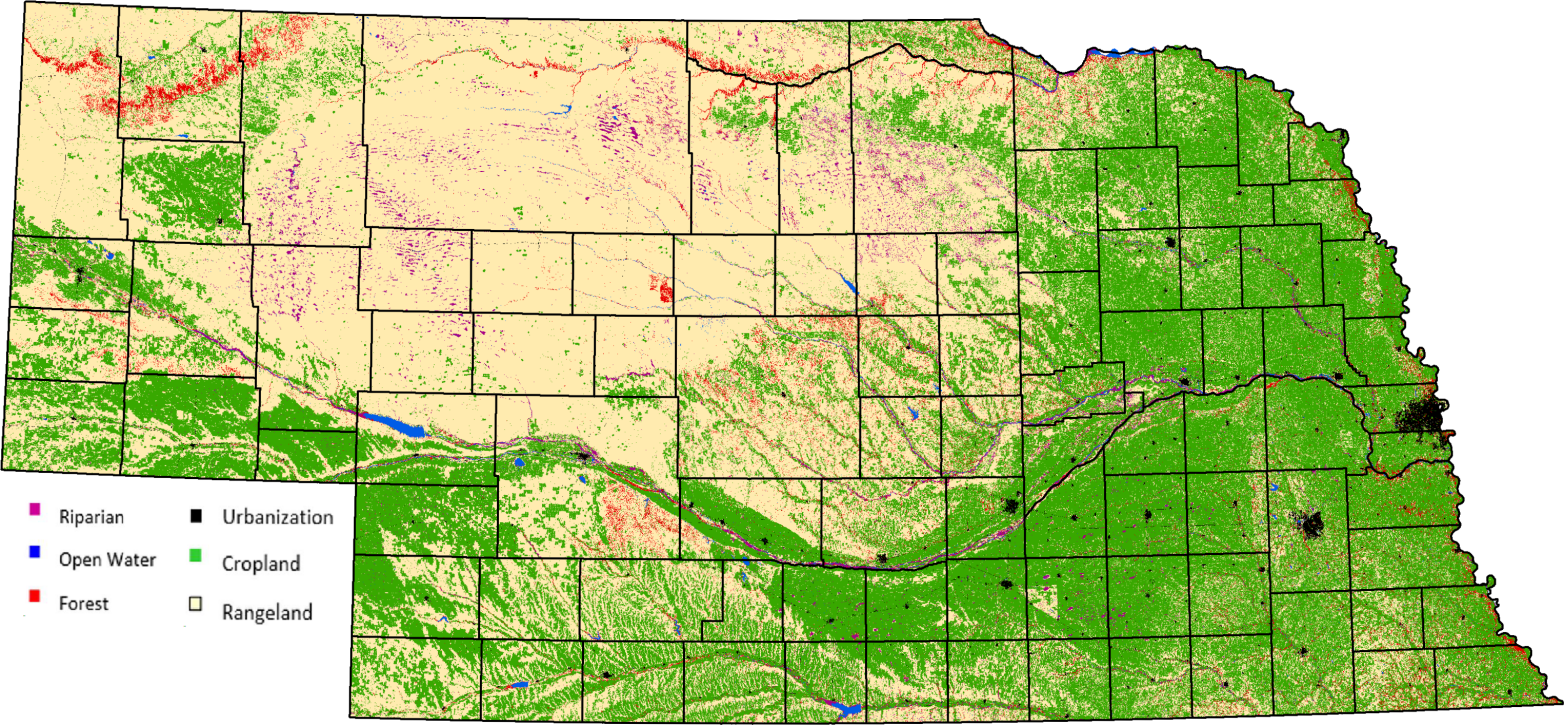
Nebraska landscape features.

### Data collection

During the nine-day November firearm season, hunters are required to present all harvested deer at one of 119 NGPC check stations across the state. NGPC employees and other check station attendants record the following data for each harvested deer: species (MD or white-tailed deer), county of kill, date of kill, days hunted, etc. All data recorded was summarized by county. While the ‘days hunted’ is a useful metric of hunter effort, which is known to influence deer harvest (Bhandari et al., 2006), this data was only collected on harvested deer and as such is a potentially biased estimate of overall hunter effort in the area, as counties with no MD harvest would also show as having no hunter effort, not because there were no hunters in the area, but only because there was no MD harvest. However, given the potential importance of hunter effort on overall harvest and the lack of unbiased data, we felt it important to include despite these concerns. Also, using the total area of each county collected from a Geographic Information System (GIS), we determined the harvest density (number of MD harvested per 100 km^2^) for each county, to control for differences in county size.

**Table 1:** The covariates that were included in the analysis of mule deer harvest density (harvest/100 km^2^) in Nebraska, USA, 2014–1016, along with the functional forms considered and included in the final analysis. Any forms that were within two AIC_c_ units of the top form were included in the analysis to determine the top model.

Covariates	**Functional forms**	
	**Considered**	**Included**
Agriculture	Linear, Pseudothreshold, Quadratic	Quadratic
Canopy cover	Linear and Pseudothreshold	Linear
Development	Linear and Pseudothreshold	Linear
Elevation	Linear, Pseudothreshold, Quadratic	Quadratic
Forest	Linear and Pseudothreshold	Linear
Hunter effort	Linear, Pseudothreshold, Quadratic	Pseudothreshold and Quadratic
NDVI amplitude	Linear, Pseudothreshold, Quadratic	Quadratic
NDVI time integrated	Linear, Pseudothreshold, Quadratic	Quadratic
Range	Linear, Pseudothreshold, Quadratic	Quadratic
Riparian	Linear and Pseudothreshold	Linear and Pseudothreshold
Road density	Linear, Pseudothreshold, Quadratic	Quadratic
Roughness	Linear, Pseudothreshold, Quadratic	Pseudothreshold
Slope	Linear, Pseudothreshold, Quadratic	Pseudothreshold and Quadratic
Urbanization	Linear, Pseudothreshold, Quadratic	Linear
Year	Categorical	Categorical

We used GIS layers to estimate different environmental and anthropogenic factors at a county level throughout the state, see Table 1. Time integrated NDVI and NDVI amplitude (USGS, 2015) both use satellite imagery to produce measures of vegetation ‘greenness’. This provides an approximation of forage availability for ungulates (Pettorelli et al., 2011), particularly in open habitats. Canopy cover was used to determine the amount of tree cover and as an additional means of measuring riparian habitat within each county (Homer et al., 2015). Additionally, the Pine Ridge, in the northwestern portion of the state, has an abundance of trees, so forested habitat was examined to include this area and others with trees that were not included within the riparian category. Percent development measures the amount of human development on the landscape, which we used to determine the amount of urbanization (Xian et al., 2011). Percent development and urbanization are correlated with road density, which likely indicates an increase in fragmentation and less habitat for MD as these covariates increase. To determine differences in the terrain, we used terrain roughness, which analyzes the change in elevation of one point in reference to its neighboring points (Sappington, Longshore & Thompson, 2007). Road density (Hayes, 2011), elevation (USGS, 2017), and slope (generated from the elevation layer in ArcGIS) were also included. Categorical variables representing forest, rangeland, agriculture, riparian, and urbanization were also included, using the percentage of the county covered by each type.

### Data analysis

We fit multiple generalized linear models with a Gamma link function using ‘glm’ in R version 3.3.2 (R Core Team, 2016), to determine the impact of 13 environmental and anthropogenic landscape features on MD harvest densities in Nebraska from 2014-2016 (Table 1). We determined the mean value of each covariate at the county scale using ArcMap 10.4, as this was the finest scale at which the harvest data was available. Given that the relationship between harvest and our covariates may be nonlinear, we evaluated multiple functional forms (linear, quadratic, pseudothreshold) for each continuous covariate in our analysis, unless the most appropriate functional form could be identified *a priori* from the existing literature (Table 1). We fit the pseudothreshold functional form using a natural log transformation (Franklin et al., 2000). Additionally, we standardized all continuous covariates by subtracting the mean and dividing by two times the standard deviation (Gelman, 2008; Lele, 2009) to allow for direct comparison of the relative importance of each covariate in our models.

We used a multi-tiered approach to model selection (Franklin et al., 2000) to reduce the number of competing models (Burnham & Anderson, 2002). Tier one was an exploratory analysis of the selected functional forms (linear, quadratic, and/or pseudothreshold) for each covariate. We ranked the resulting models for each covariate using AIC_c_ and advanced those functional forms that were within 2 AIC_c_ of the top functional form to the next tier, following Ranglack et al. (2017). In tier two, we combined the top functional form of each covariate in all possible combinations to determine the best-supported model for MD harvest in Nebraska. As this was the first step where we included multiple covariates in a single model, we screened all covariates for multi-collinearity using Pearson’s correlations coefficients, using —0.6— as a basis of determining correlation. Therefore, any covariates that were found to be collinear were not included in the same model, but both were examined in the absence of the other. We removed uninformative covariates following the recommendations Arnold (2010), when necessary. Year was also included as a categorical variable to examine any potential differences between years. Finally, we ranked the resulting models using AIC_c_ to determine the most supported model predicting MD harvest in Nebraska.

We validated our top model of MD harvest in Nebraska using a *k*-fold temporal cross-validation, to determine the temporal predictability of the model (Boyce et al., 2002; Wiens et al., 2008). We used two of the three years to train the model and predict MD harvest of the remaining year. This was repeated such that each year was predicted by the other two. We then used Spearman’s rank correlation (Boyce et al., 2002) to compare the predicted and actual harvest densities for each temporal fold and used the average Spearman’s rank correlation to determine overall model validity.

## Results

**Figure 2:**
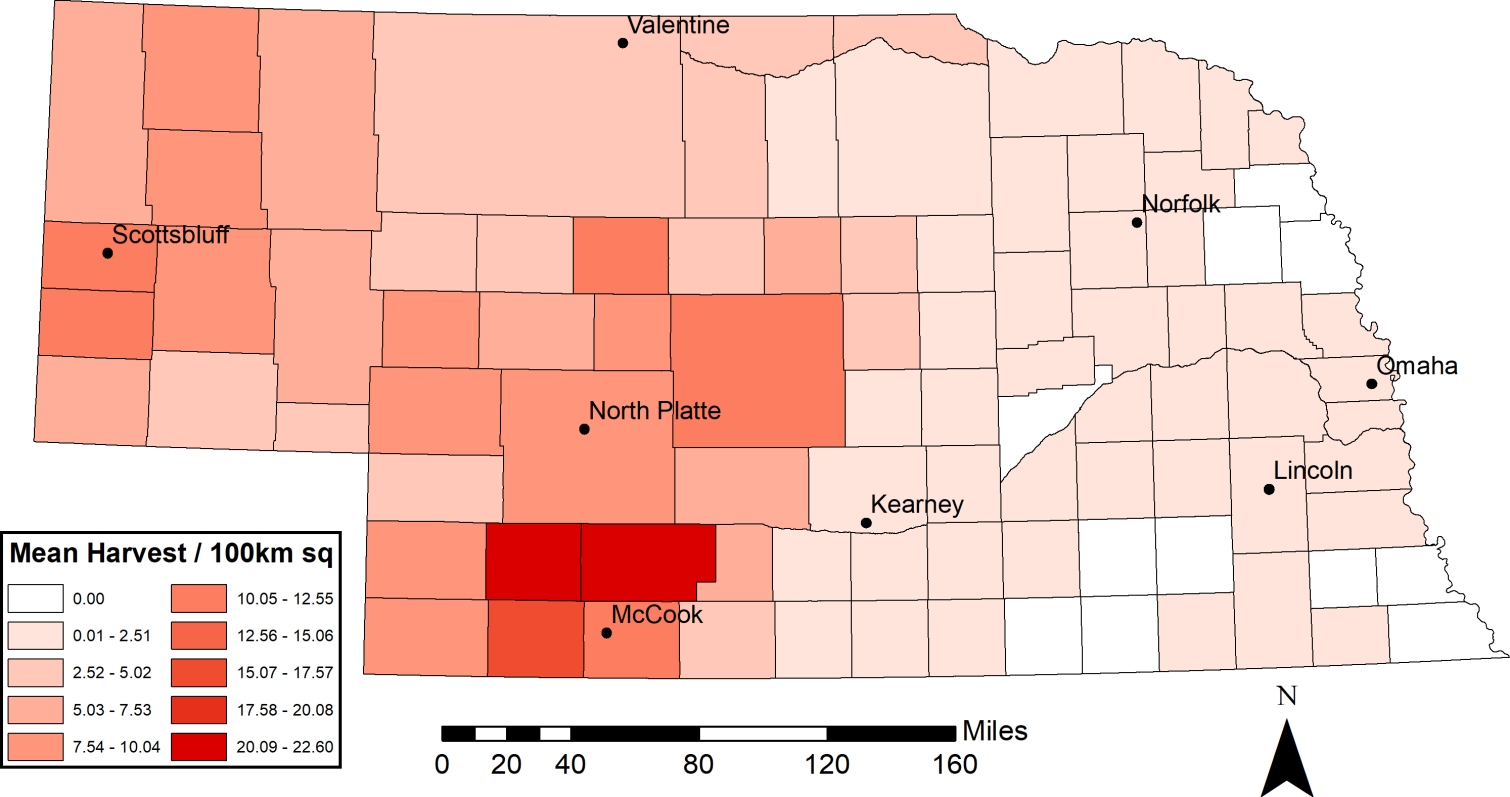
Mean actual mule deer harvest density (individuals/100 km^2^) from 2014–2016 in Nebraska, USA by county.

Our dataset contained a total of 26,255 harvested MD from three rifle deer seasons (2014–2016). Most counties (82 of 93) reported MD harvest during the three-year period. The mean harvest density for the 82 counties was 3.79 MD harvested/100 km^2^, with a range of 0–22.60 MD harvested/ 100 km^2^ (Fig. 2 and Table S1).

**Figure 3:**
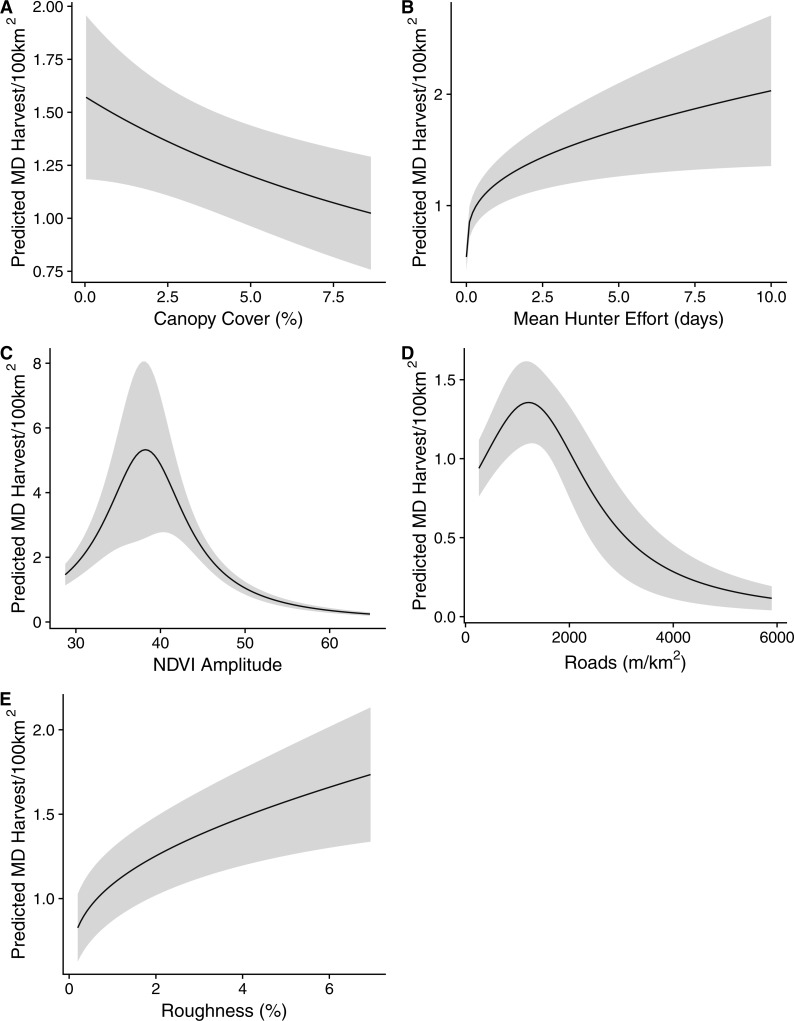
Plots of the five covariates included in the top model of mule deer (MD) harvest density (individuals/100 km^2^) in Nebraska, USA, 2014–2016, on the original, non-standardized scale. The black lines represent the coefficient estimate and the shaded areas represent the 95% confidence interval across the available range of each covariate, while the other covariates were held at their mean value. (A) Canopy cover; (B) Mean hunter effort; (C) NDVI amplitude; (D) roads; (E) roughness.

**Figure 4:**
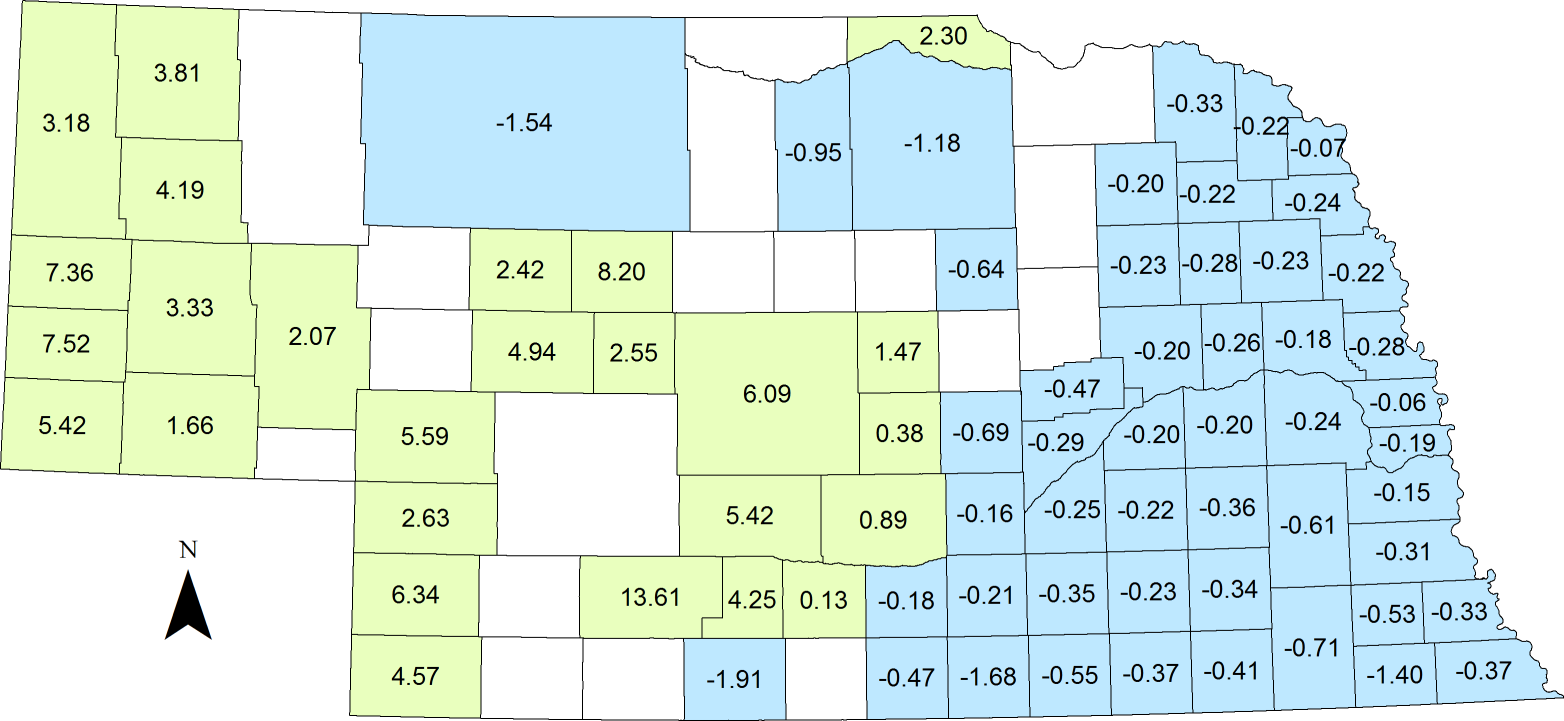
Differences between mean actual and predicted mule deer harvest density (individuals/100 km ^2^) from our top model Counties in blue indicate that actual harvest was significantly less than predicted, while counties in green indicate that actual harvest was significantly more than predicted. Residual values (actual—predicted harvest density) are provided for those counties where there were significant differences.

Tier one of our analysis determined our top functional forms for each covariate (Table 1). The top model produced during the second tier of our analysis consisted of the covariates, in order of relative importance: mean NDVI amplitude, hunter effort, mean road density, mean roughness, and mean percent canopy cover (Table 2). Percent canopy cover showed a negative relationship with harvest density (Fig. 3). Mean NDVI amplitude and mean road density both produced convex quadratic relationships with MD harvest densities. Optimal harvest was achieved in areas where time integrated NDVI was ∼38, and areas where road density was 1,750 m/km^2^ (Fig. 3). A convex pseudothreshold relationship was most supported for hunter effort terrain roughness, and the greatest harvest densities were recorded when mean hunter effort and roughness was highest (Fig. 3). During the validation of our model, we recorded a mean Spearman’s rank correlation coefficient value = 0.753, and a mean *p*-value < 0.001 (Table 3). Our top model predicted a mean of 2.62 MD harvested/100 km^2^, and a range of 0.10–27.51 MD harvested/100 km^2^. Of the 93 counties in Nebraska, mean actual harvest over the 3 year period of our study was significantly greater than predicted in 26 counties, and significantly lower than predicted in 49 counties, however, these differences were generally small (Fig. 4).

**Table 2:** The functional form, standardized coefficient estimate, and standard error of covariates included in top model of mule deer harvest (individuals/100 km^2^) in Nebraska, 2014–2016.

**Covariate**	**Function form**	**Estimate**	**Std. error**
Intercept		1.04	0.09
Canopy cover	Linear	0.18	0.08
Hunter effort	Pseudothreshold	−0.99	0.17
NDVI amplitude	Linear	2.19	0.28
	Quadratic	2.16	0.24
Roads	Linear	0.14	0.16
	Quadratic	0.79	0.26
Roughness	Pseudothreshold	−0.29	0.07

**Table 3:** Spearman rank correlation coefficient and p-values from the temporal *k*-folds cross validation of our top model of mule deer harvest in Nebraska, USA, 2014–2016. The data presented indicate which year was being used as a validation dataset.

Test	2014	2015	2016	Mean
Spearman rank	0.573	0.854	0.831	0.753
*p*-value	<0.01	<0.01	<0.01	<0.01

## Discussion

Our results suggest that resources for MD management should be focused on areas of decreasing canopy cover, increasing terrain roughness, road densities less than 2,000 m/km^2^, increasing hunter effort, and NDVI amplitude values around 38 (Fig. 3), if the management goal is higher hunter harvest. As an index of forage quality, NDVI has become a very useful tool in wildlife ecology and management (Pettorelli et al., 2011) and was the most important covariate in our top model. Though additional field data is often required to fully understand the relationship between forage quality and NDVI (Borowik et al., 2013), in our case, we do not believe that NDVI is best used as a surrogate for forage availability, but rather as a continuous measure of land use (Bradley & Mustard, 2007). The NDVI amplitude value of 38 roughly correlates with rangeland habitat, with agricultural and forested lands showing significantly higher NDVI values due to higher water availability.

Hunter effort was the second most important covariate in our top model. This is expected, as hunter effort and motivation is known to influence harvest rates (Bhandari et al., 2006). However, it is important to note that our hunter effort data only included the number of days hunted by hunters who successfully harvest MD in each county. This does not include the large numbers of whitetail deer hunters, or those hunters who were unsuccessful. The addition of hunter effort for both successful and unsuccessful hunters should be a focus of future research and may reduce the difference between predicted and actual harvest.

Road density was the next most important covariate in our top model, which we believe to be a factor of accessibility and disturbance. Harvest reached a peak at about 1,750 m/km^2^ which indicates there are enough roads to allow hunters the access needed to find and get to the deer, while not causing enough disturbance to deter deer from using the area. Areas with lower road densities are likely to have just as good or better MD habitat, but do not allow enough access for hunters to be as successful in harvesting MD. However, areas with higher road densities are likely poor-quality habitats due to the increased amount of disturbance and fragmentation (Sawyer et al., 2006; Sawyer et al., 2007; Rogala et al., 2011).

Harvest also increased with increased terrain roughness. Mackie (1970) found that MD prefer areas with increased slope and terrain roughness, which are not likely to be urbanized or developed and consequently more likely to be rangeland dominant. This likely contributes to the increased harvest density of MD as terrain roughness increases. Therefore, rangeland habitat in Nebraska is important for MD, as shown by both NDVI and terrain roughness.

The decrease in harvest densities as canopy cover increases is likely due to influences on the hunters’ ability to access and locate deer, rather than habitat quality. Forested areas are likely to be less fragmented, making hunter accessibility more difficult; therefore, the ability of hunters to navigate through the terrain likely decreases, along with visibility, which in return decreases harvest (Brinkman et al., 2009). Alternatively, MD in Nebraska may avoid forested areas, leading to lower harvest in those areas. This may be due to competition with whitetail deer and disturbance. The majority of forested habitat in Nebraska are riparian areas surrounded by agriculture and urbanization or development. These fragmented agricultural habitats generally support high densities of whitetail populations (Lingle, 2002), which prefer gentle terrains generally consisting of agricultural lands. Further research on MD habitat selection is needed to qualify the importance of this covariate.

The differences between our actual and predicted harvest (Fig. 4) is likely due to limiting factors during harvest that could not be included in the model, as harvest is not a random process, but is skewed by law, hunter behavior, hunter access, and other factors. While statewide MD population estimates are not available at the county scale, in the southeastern portion of the state, MD population sizes are very small, thus we are seeing lower harvest than expected. Likewise, in the western and central portions of the state there are very strong MD populations, which can help to explain the higher than expected harvest. The inclusion of county level MD population estimates would likely explain more of the variation in our data and improve model fit. Additionally, Mysterud (2011) showed that large mammal hunters have many factors influencing selectivity during hunts, including: individual preference, opportunity, regulations, hunting styles, population structure, and animal abundance. The type of hunter (trophy or meat, local or foreign) also influences the likelihood of harvest (Mysterud, Tryjanowski & Panek, 2006; Mysterud, 2011). Finally, Nebraska is also 97% privately owned, and therefore, hunters are not capable of accessing all available land that MD prefer, which likely causes a decrease in harvest due to lack of public access.

Given that Nebraska is largely a privately-owned state, incentives for landowner conservation may be needed, such as the Grassland Reserve Program (GRP), the Conservation Reserve Program (CRP) (USDA, 2010; USDA, 2016), or a community-based wildlife management scheme (Ranglack & Toit, 2015). Deutsch (2009) showed that the involvement of local communities helps increase the success of rangeland wildlife conservation, which could be very beneficial in areas of the state under high demand for MD conservation. Community involvement funds could also be given as local scholarships or for community development or projects (Frost & Bond, 2008). This would likely increase the amount of land available to public hunters and could reduce the difference between actual and predicted harvest.

## Conclusions

The understanding of habitat types that lead to higher MD harvest density can be beneficial to management, as areas that have higher harvest densities are likely correlated to having higher MD populations. This is a key piece of information for wildlife managers for effective resource management. In Nebraska, efforts are being made to increase MD populations, and understanding their habitat choices would allow managers to effectively target their management actions. Our results suggest that conservation efforts should be allocated to areas matching the description of our top four habitat covariates (NDVI amplitude values near 38, road density near 1,750 m/km^2^, increasing terrain roughness, and decreasing canopy cover). These areas have the capabilities for producing the highest harvest densities for MD, and likely indicate better quality habitat. This is not likely the best quality habitat for MD due to the presence of moderate road densities, but the increasing harvest densities shows that the habitat is suitable for MD. The best quality habitat is likely in areas farther from roads (Sawyer et al., 2006; Sawyer et al., 2007; Rogala et al., 2011; Ranglack et al., 2016), but further research is needed into MD habitat selection to fully understand the impacts of our top four habitat covariates on habitat selection. Additionally, hunter effort can be managed through the use of shorter or longer seasons to decrease or increase the number of MD harvested respectively, depending on management goals. This allows for scientifically informed management of MD, their habitat, and their harvest.

##  Supplemental Information

10.7717/peerj.5510/supp-1 Supplemental Information 1 Mule deer harvest densities (harvest/100 km ^2^) for each county during each year, along with the mean harvest density

10.7717/peerj.5510/supp-2 Data S1 Raw data for analysis

10.7717/peerj.5510/supp-3 Supplemental Information 2 Code used for analysis

## Additional Information and Declarations

### Data Availability

The following information was supplied regarding data availability: The raw data and code are included as Supplemental Files.
