# Supplementary material for: Effects of environmental and anthropogenic landscape features on mule deer harvest in Nebraska
Source: PeerJ. 2018 Sep 10;6:e5510. doi: 10.7717/peerj.5510 (PMC6136395; doi:10.7717/peerj.5510)
Supplement: Supplemental Information 1 [file peerj-06-5510-s001.docx]

**Supplemental Material**

**Table S1.** Mule deer harvest densities (harvest / 100 km^2^) for each county during each year, along with the mean harvest density.

| County | Mule Deer Harvest / 100km^2^ | | | |
| --- | --- | --- | --- | --- |
|  | **2014** | **2015** | **2016** | **Mean** |
| ADAMS | 0.00 | 0.07 | 0.07 | 0.05 |
| ANTELOPE | 0.45 | 0.68 | 0.32 | 0.48 |
| ARTHUR | 6.72 | 8.50 | 7.91 | 7.71 |
| BANNER | 9.63 | 11.54 | 12.17 | 11.11 |
| BLAINE | 4.27 | 4.98 | 3.62 | 4.29 |
| BOONE | 0.62 | 0.62 | 0.79 | 0.68 |
| BOX BUTTE | 7.10 | 7.45 | 9.00 | 7.85 |
| BOYD | 4.33 | 5.11 | 4.25 | 4.56 |
| BROWN | 2.21 | 2.87 | 3.28 | 2.79 |
| BUFFALO | 1.11 | 2.02 | 1.66 | 1.60 |
| BURT | 0.00 | 0.00 | 0.00 | 0.00 |
| BUTLER | 0.07 | 0.13 | 0.00 | 0.07 |
| CASS | 0.07 | 0.20 | 0.14 | 0.14 |
| CEDAR | 0.10 | 0.05 | 0.05 | 0.07 |
| CHASE | 8.65 | 8.99 | 10.89 | 9.51 |
| CHERRY | 3.19 | 3.43 | 3.91 | 3.51 |
| CHEYENNE | 4.13 | 4.91 | 5.91 | 4.98 |
| CLAY | 0.00 | 0.00 | 0.07 | 0.02 |
| COLFAX | 0.00 | 0.00 | 0.18 | 0.06 |
| CUMING | 0.00 | 0.00 | 0.00 | 0.00 |
| CUSTER | 10.36 | 12.25 | 11.16 | 11.26 |
| DAKOTA | 0.14 | 0.29 | 0.00 | 0.14 |
| DAWES | 7.66 | 9.32 | 10.42 | 9.13 |
| DAWSON | 5.72 | 6.75 | 7.05 | 6.51 |
| DEUEL | 2.54 | 2.72 | 3.16 | 2.81 |
| DIXON | 0.00 | 0.08 | 0.00 | 0.03 |
| DODGE | 0.07 | 0.00 | 0.07 | 0.05 |
| DOUGLAS | 0.00 | 0.11 | 0.00 | 0.04 |
| DUNDY | 9.27 | 9.56 | 9.31 | 9.38 |
| FILLMORE | 0.00 | 0.00 | 0.00 | 0.00 |
| FRANKLIN | 0.00 | 0.40 | 0.00 | 0.13 |
| FRONTIER | 22.11 | 22.94 | 22.74 | 22.60 |
| FURNAS | 3.22 | 2.84 | 3.81 | 3.29 |
| GAGE | 0.09 | 0.00 | 0.04 | 0.04 |
| GARDEN | 5.38 | 6.85 | 6.76 | 6.33 |
| GARFIELD | 4.87 | 4.06 | 3.52 | 4.15 |
| GOSPER | 5.26 | 5.59 | 5.68 | 5.51 |
| GRANT | 3.11 | 4.69 | 5.43 | 4.41 |
| GREELEY | 1.76 | 1.35 | 2.30 | 1.80 |
| HALL | 0.07 | 0.07 | 0.00 | 0.05 |
| HAMILTON | 0.00 | 0.00 | 0.07 | 0.02 |
| HARLAN | 0.87 | 0.74 | 0.67 | 0.76 |
| HAYES | 19.66 | 21.55 | 23.12 | 21.44 |
| HITCHCOCK | 15.69 | 15.96 | 18.49 | 16.71 |
| HOLT | 0.48 | 0.73 | 0.59 | 0.60 |
| HOOKER | 3.53 | 3.64 | 5.19 | 4.12 |
| HOWARD | 0.34 | 0.47 | 0.34 | 0.38 |
| JEFFERSON | 0.07 | 0.00 | 0.00 | 0.02 |
| JOHNSON | 0.00 | 0.00 | 0.00 | 0.00 |
| KEARNEY | 0.07 | 0.07 | 0.00 | 0.05 |
| KEITH | 6.89 | 7.73 | 8.74 | 7.79 |
| KEYA PAHA | 2.09 | 4.99 | 3.39 | 3.49 |
| KIMBALL | 6.65 | 6.45 | 7.06 | 6.72 |
| KNOX | 0.75 | 0.54 | 0.61 | 0.63 |
| LANCASTER | 0.09 | 0.05 | 0.05 | 0.06 |
| LINCOLN | 9.08 | 9.78 | 10.20 | 9.69 |
| LOGAN | 9.27 | 8.93 | 9.61 | 9.27 |
| LOUP | 6.09 | 6.50 | 6.43 | 6.34 |
| MADISON | 0.20 | 0.20 | 0.07 | 0.16 |
| MCPHERSON | 7.46 | 7.64 | 6.42 | 7.17 |
| MERRICK | 0.00 | 0.00 | 0.00 | 0.00 |
| MORRILL | 9.48 | 10.43 | 10.16 | 10.02 |
| NANCE | 0.26 | 0.43 | 0.09 | 0.26 |
| NEMAHA | 0.00 | 0.00 | 0.00 | 0.00 |
| NUCKOLLS | 0.00 | 0.00 | 0.00 | 0.00 |
| OTOE | 0.06 | 0.06 | 0.00 | 0.04 |
| PAWNEE | 0.18 | 0.00 | 0.09 | 0.09 |
| PERKINS | 3.67 | 4.11 | 5.29 | 4.36 |
| PHELPS | 0.64 | 0.36 | 0.36 | 0.45 |
| PIERCE | 0.13 | 0.27 | 0.13 | 0.18 |
| PLATTE | 0.06 | 0.00 | 0.17 | 0.08 |
| POLK | 0.09 | 0.00 | 0.00 | 0.03 |
| RED WILLOW | 9.57 | 9.79 | 11.83 | 10.40 |
| RICHARDSON | 0.00 | 0.00 | 0.00 | 0.00 |
| ROCK | 1.57 | 1.53 | 1.30 | 1.47 |
| SALINE | 0.00 | 0.00 | 0.00 | 0.00 |
| SARPY | 0.00 | 0.00 | 0.16 | 0.05 |
| SAUNDERS | 0.05 | 0.00 | 0.00 | 0.02 |
| SCOTTS BLUFF | 11.14 | 11.35 | 13.42 | 11.97 |
| SEWARD | 0.00 | 0.07 | 0.07 | 0.05 |
| SHERIDAN | 5.54 | 6.19 | 6.97 | 6.23 |
| SHERMAN | 1.42 | 2.16 | 1.69 | 1.76 |
| SIOUX | 4.13 | 5.01 | 5.98 | 5.04 |
| STANTON | 0.00 | 0.18 | 0.00 | 0.06 |
| THAYER | 0.00 | 0.00 | 0.00 | 0.00 |
| THOMAS | 7.96 | 12.56 | 11.04 | 10.52 |
| THURSTON | 0.00 | 0.00 | 0.00 | 0.00 |
| VALLEY | 2.84 | 3.93 | 3.12 | 3.30 |
| WASHINGTON | 0.10 | 0.00 | 0.20 | 0.10 |
| WAYNE | 0.09 | 0.00 | 0.00 | 0.03 |
| WEBSTER | 0.13 | 0.07 | 0.13 | 0.11 |
| WHEELER | 1.75 | 1.81 | 1.54 | 1.70 |
| YORK | 0.07 | 0.00 | 0.07 | 0.05 |
